# The development of the soderberg electrolyzer electromagnetic field’s state monitoring system

**DOI:** 10.1038/s41598-024-52002-w

**Published:** 2024-02-12

**Authors:** Ilyushin Yury, Alexander Martirosyan

**Affiliations:** Saint Petersburg Mining University, Saint Petersburg, Russia 199106

**Keywords:** Computer science, Software, Electrical and electronic engineering

## Abstract

This study is devoted to improving the economic efficiency of the cell, due to the field of the generated electromagnetic field’s accurate diagnostics. To solve this problem, the authors had developed a hardware-software complex for electromagnetic field diagnostics. This complex includes a measurement device and a software package for data collection and analysis. On the laboratory prototype of the aluminum electrolysis complex, a study was carried out on the formation and structure of the electromagnetic field. A number of experiments have been carried out showing the degree of formation of the electromagnetic field by the anode, the relationship of electromagnetic fields in the inter-anode space has been shown. Based on the results of the studies, conclusions were drawn about the possibility of diagnosing the current state of the anode, determining the direction of rotation of aluminum in the electrolytic cell and estimating the life of the anode and its burnout time.

## Introduction

In November 2022, the United Nations announced that the world's population had crossed the threshold of 8 billion people^1^. Constantly growing market needs of the vehicles, consumer goods, packaging, construction, packaging materials, etc. needs the increasing of the burden on the metallurgical industry. The volume of the most used metals extraction (lead, aluminum, zinc, etc.) is no longer enough to meet all the needs of the industry and the market. The review of BCS analysts notes that the shortage of aluminum in the world will remain a key factor in supporting metal prices and may lead to their increase^2,3^. According to them, in the third quarter of 2022, the deficit of aluminum in the world amounted to 1.1 million tons^4^. The average selling price of aluminum in the first half of 2022 was USD 3,365/t (+ 47.1% YOY), supported by an increase in the average price on the London Metal Exchange taking into account the quotation period (LME QP) 1 (+ 45, 1% YoY, up to $3,023/t). At the same time, aluminum prices increased significantly in 1Q 2022, reaching USD 3,985. The cost of aluminum production in the first half of 2022 increased by 33.2% to USD 2,028 per ton (compared to USD 1,523 US dollars per tonne in the first half of 2021) due to increased costs for alumina, other raw materials and materials, as well as logistics^5^. An important factor in the pricing of primary aluminum is the efficiency of aluminum production. According to metallurgical companies, the cost of primary aluminum is growing by 5–10 percent annually. This is mainly due to the increase in the price of electricity, which is the most important and most energy-intensive resource (up to 65% of the cost of primary aluminum). Thus, the analysis of the technological process of obtaining primary aluminum, the identification of the most energy-consuming factors and their optimization will significantly reduce the cost of the final product.

## Methods

### Description of the technological process

Modern industrial production of aluminum is based on the electrolytic decomposition of alumina (Al_2_O_3_) in the melt of cryolite salts (3NaF∙AlF3). This production technology can be provided by several types of electrolyzers. Prebaked anode (BA) cells operate at over 320 kA, and prototype designs over 400 kA, and 500 kA designs are reportedly under development. The productivity of modern electrolyzers reaches 2500 kg per day^6^.

The cathode device of the ensouled anode cell (EAC) electrolyzer is a powerful metal cathode casing, in which a carbon conductive bath about 50 cm deep is located, isolated from the casing by a refractory and heat-insulating lining. Liquid aluminum accumulates at the bottom of the bath shaft, and an electrolyte layer is located above it, in which the anode is immersed.

The anode device consists of carbon anode blocks arranged in two rows. The current is supplied to them through an anode rod attached to the anode bus. The escaping anode gases are collected in the gas collection casing and sucked off by smoke exhausters to the gas treatment plants.

Electrolyzers with Soderberg anodes (ESA) differ from EAC electrolyzers in the design of the anode. Pre-baked coal blocks are installed on the EAC electrolyzers, and on the ESA electrolyzers, the anode is formed from the carbonaceous mass directly on the electrolyzer under the action of the released heat. The designs of the cathode device for electrolyzers of all types do not have significant differences.

**Figure 1:**
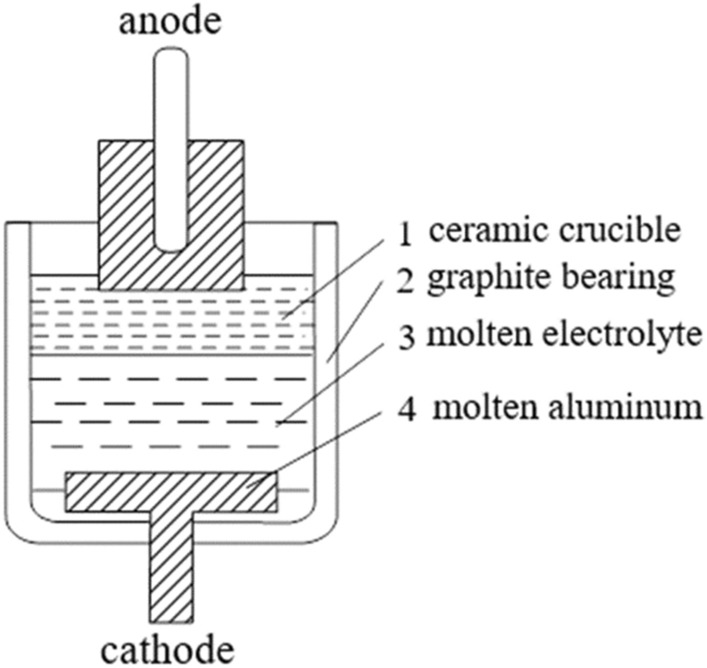
Diagram of an electrolytic cell.

Theoretically, it is convenient to consider the process of aluminum electrolysis on the diagram of the simplest electrolytic installation (Fig. 1).

An anode is located in the upper part of the cell, partially immersed in the molten electrolyte 3. When the positive pole of the DC source is connected to the anode and the negative pole to the graphite plate, an electric current will flow through the cell, which will cause chemical changes in the electrolyte. This process is called electrolysis. During the decomposition of alumina, aluminum is released at the cathode, and oxygen is released at the anode, which, oxidizing the carbon anode, leads to the formation of CO and CO_2_.

In order for an electric current to flow through a cell, a certain voltage must be applied to it. If this voltage is greater than the alumina decomposition voltage Ep, then the electrolysis process will begin. If the applied voltage is less than Ep, no current will flow through the cell.

With an inert anode (platinum, ferrites, etc.), the decomposition of alumina will occur according to the reaction:

$${\text{Al}}_{{2}} {\text{O}}_{{3}} \leftrightarrow {\text{ 2Al }} + { 1}.{\text{5O}}_{{2}} \;{\text{while}}\;{\text{ E}}_{{\text{p}}} = { 2}.{\text{19 V }}\left[ {{6},{ 8},{ 9}} \right].$$

On the carbon anode, which is equipped with all operating electrolyzers, two reactions are possible—with the formation of CO2 and with the formation of CO:

$${\text{2Al}}_{{2}} {\text{O}}_{{3}} + {\text{ 3C }} \leftrightarrow {\text{ 4Al }} + {\text{ 3CO}}_{{2}} \;{\text{while}}\;{\text{Ep }} = { 1}.{\text{167 V}};$$

$${\text{Al}}_{{2}} {\text{O}}_{{3}} + {\text{ 3C }} \leftrightarrow {\text{ 2Al }} + {\text{ 3CO}}\;{\text{while }}\;{\text{E}}_{{\text{p}}} = { 1}.0{34}\;{\text{ V }}\left[ {{7},{ 24},{ 25}} \right].$$

That is, with a carbon anode, the voltage of alumina decomposition is less than with an inert anode, since part of the energy necessary for the decomposition of alumina is released during the oxidation of the carbon anode. Therefore, the minimum voltage at which the electrolysis process begins is commonly called the back EMF.

Measurements carried out on industrial electrolyzers showed that back EMF is in the range of 1.4–1.8 V, and its value is influenced by the anode current density, the chemical activity of the carbon anode, the process temperature, and a number of other factors^3^. Consider the main parameters of the electrolysis process.

The amount of aluminum Q_o_ (kg), which theoretically can be obtained in the electrolysis process for a certain period of time t (h), is determined by Faraday's law Q_o_ = k∙I∙t, where k is the electrochemical equivalent of aluminum, equal to 0.3354 g/A∙h or kg/kA∙h; I is the current strength, kA.

In practice, due to the oxidation reactions of the obtained aluminum in the electrolyte during its interaction with the anode gases, as well as due to some current leakage, the actual amount of aluminum obtained Q_a_ is always less than the theoretically possible value. The ratio of the practically obtained metal Q_a_ to the theoretically possible Q_o_ is called the current output η and is expressed as a percentage or fractions of a unit:

$$\upeta = \frac{{Q}_{o}}{{Q}_{a}}$$

Then

$${Q}_{a} =\mathrm{ k}\cdot {\text{I}}\cdot {\text{t}}\cdot\upeta .$$

The current output characterizes the efficiency of current use and is the most important technical indicator of the electrolysis process. On modern electrolyzers, the value of η is in the range from 0.85 to 0.95. The daily capacity of the Q_24_ cell (which in practice is called "bath-day") is equal to

$${{\text{Q}}}_{24} =\mathrm{ k}\cdot {\text{I}}\cdot {\text{t}}\cdot\upeta =\mathrm{ 0,3354}\cdot 24\cdot {\text{I}}\cdot\upeta =\mathrm{ 8,05}\cdot {\text{I}}\cdot\upeta .$$

### Specific electricity consumption

A very important indicator of the efficiency of electrolysis technology is the specific consumption of direct current electricity per unit of aluminum produced w (kW h / t), that is, the ratio of the energy expended W (kW h) to the amount of aluminum produced Q_a_ (t). The amount of energy spent W at direct current is determined by the formula

$$\mathrm{W }=\mathrm{ I}\cdot {{\text{U}}}_{{\text{m}}}$$

where ${\text{I}}$ is the current strength, kA; ${{\text{U}}}_{{\text{m}}}$ is the average voltage on the cell, V; $t$ is the duration of the electrolysis process, h.

The specific consumption of direct current electricity for the production of 1 ton of aluminum is calculated by the formula:

$$\mathrm{W }=\frac{W}{O}=\frac{I\cdot {U}_{m}\cdot t}{k\cdot I\cdot t\cdot\upeta }=\frac{{U}_{m}}{k\cdot\upeta }$$

The specific power consumption at operating electrolyzers is 13.5–16 thousand kWh/t. In practice, the reciprocal of the specific electricity consumption is often used—energy efficiency (${\upeta }_{e}$), this is the aluminum yield in grams per kWh consumed:

$${\upeta }_{e} =\frac{1}{\upeta }=\frac{k\cdot\upeta }{{U}_{m}}$$

When converting alternating current to direct current, power losses are formed in the busbar, as well as in transformers and rectifiers of converter units. The conversion factor (efficiency factor) of silicon-converting substations (SCS) of aluminum plants is an integral indicator that characterizes the amount of electrical energy loss for converting industrial frequency alternating current into rectified current. The coefficient is determined by calculation at the design stage of the object. In general, it depends on the following parameters:

1. Gearbox equipment layout, voltage class, rectified current value. At AZ ADV, substations with a rectified voltage of 450, 600, 950, 1000 and 1500 V are in operation. The magnitude of the rectified current is from 140 to 313 kA.
2. The number of rectifier units installed at one SCS, their technical characteristics.
3. Cross-sections and lengths of current-carrying conductors of the power supply circuit (losses are directly proportional to the square of the current multiplied by the resistance of the conductor).
4. Accuracy class and type of rectified current measurement systems (measurement system IS-225, monoblock high current meter PIPT-180, FOCS-350).
5. Repair modes of the equipment’s operation of the SCS and GPP, the withdrawal of individual elements of the equipment from work during the modernization of facilities.
6. Temperature regimes of outdoor air.

The conversion factor is an important indicator characterizing the losses of the SCS, which is controlled, calculated and taken into account on a daily basis. Changing the conversion factor makes it possible to improve the accuracy of determining the state of the rectified and commercial current measurement systems, changing the power supply circuits of the SCS during the day, and the operation of the equipment in repair mode. The maintenance of technological and energy parameters (current) of the electrolysis series is carried out by switching the tap changer stages of the transformers or by changing the current saturation chokes. In this case, the conversion factor changes. Which allows you to calculate the weighted average daily.

With long-term operation of the equipment and a gradual change in their parameters (equipment aging), the losses increase, while the conversion factor decreases.

These conversion losses are determined by calculation, and the consumption of energy for the own needs of the converter substation is added to them. The ratio of direct current energy $W$ to the received alternating current energy ${W}_{AC}$ is called the conversion factor EF (efficiency factor):

$${\text{EF}}=\frac{W}{{W}_{AC}}$$

At modern plants, the EF value is in the range of 0.96–0.98. Conversion losses also depend on the type of rectifiers and transformers used, they decrease with increasing current strength and, especially, with increasing number of operating electrolyzers in a series. The dominant part of the energy losses occurs in the transformers of the rectifier units. Energy losses in transformers consist of losses in the transformer windings (“copper losses”) and iron remagnetization losses (“iron losses”). Losses in copper reach 80–85% of the total energy losses in transformers and do not depend on the voltage value. Losses in the iron of transformers are relatively small and increase to a lesser extent with increasing voltage than losses in copper with an adequate increase in current strength.

Thus, a number of key conclusions can be drawn:

1. Electric current (electromagnetic field) is the main factor shaping the process of electrolysis and production of aluminum.
2. An electrolyser, as a complex technical device, has many technically complex elements where electrical energy losses occur, most of which can be traced only by the consequences they have on the technological process.

These conclusions, to one degree or another, were considered by scientists from many countries and pointed to the features of this technological process as an object of control. It is proposed to consider some of them.

### The state of knowledge of the problem

The first work on the interaction of the electromagnetic field dates back to 1819, in which the Danish physicist H.K. phenomena are interconnected. In 1832, Faraday established that the mass of the substance released on the electrode is directly proportional to the electric charge that has passed through the electrolyte. In 1886, Martin Hall creates the first electrolyzer. After that, many scientists around the world began to engage in research in the field of electrolysis, and the production of electrical energy in general. The main works in this area belong to Borisoglebsky Yu.V., Begunov A.I., Levin M.V., Arkhipov G.V., Vetyukov M.M., Zelberg B.I., Lokshin R.G., Kuptsov A. N., Demykin P.A., Kontsur E.P., Mintsis M.Ya., Mann V.Kh., Piskazhova T.V., Skornyakov V.I., Chalykh V.I., Polyakov P.V ., Berezin A.I., Nikolaev I.V. Platonov V.V., and many others. Research in this area abroad is carried out by a number of scientists: A. Zarouni, L. Mishra, J. Thonstad, M. Dupuis, V. Bojarevics, and others. Boikov A. in his works, for example^7,8^, shows the importance of analyzing the cell as a complex system, taking into account multiple input and output parameters. And in^9^ he uses a neural network to analyze the structure of the quality of the metal. The work^10^ considers the energy efficiency of the units and their impact on the final cost of the manufactured product. The economic assessment of general costs^11^ and the assessment of environmental pollution^12^ are rather difficult to assess. Therefore, these factors will not be assessed in the present study.

A special role should be given to the work^13^, which presents a three-dimensional non-stationary model of a 600 kA electrolytic cell. and made a detailed description of the process of electrolysis occurring in the cell. However, this mathematical model has a number of disadvantages associated with a rather large discretization of the model^14^. In order to increase the accuracy of calculations of electromagnetic fields of electrolyzers, Chinese scientists^15^ propose to use a genetic neural network. Similar conclusions were reached in works^16–21^. In general, field monitoring issues are dealt with in the context of the study of technological processes as objects of control. This approach provides high visibility, but rather low measurement accuracy. To improve the accuracy of the study, various mathematical tools are used, for example, in^22^, a new model of an uncontrolled neural network, called a network with slow functions (SFNet), is proposed for monitoring dynamic processes. Thus, the main direction of increasing the measurement accuracy is the development of new methods and models for processing the obtained models^23^. Another direction in this area is the experimental direction. The essence of this direction is to conduct a huge number of experiments and build regression models based on it^24,25^. These models are highly accurate at a given time, but are completely inapplicable to the analysis of dynamic systems with constantly changing input, internal and output parameters^26,27^. If we analyze a complex but relatively stable process, for example^28^, this approach takes place, but it is not without drawbacks, for example, in such models it is quite difficult to determine which parameters are responsible for the environment^29^, the technological process, etc. conditions when the rate of aluminum production is 6–8 h, this approach is not applicable. When analyzing such systems, it is necessary to take into account not only factors, but also the geometric parameters of the object. Such an approach in systems theory is called spatially distributed. This approach was first proposed by Pershin I.M.^29^ and continued in the works of his students^30,31^. European scientists^32,33^ also take into account spatial components when automating technological processes. Including when studying the issues of deformation of aluminum products^34–42^. This approach makes it possible to accurately determine the parameters of the technological process^43,44^. Within the framework of this study, it is proposed to consider an experiment, as a result of which, using an independently developed device, models of a dynamically changing electromagnetic field can be obtained.

Speaking about the methodological component, it is important to mention, that in modern economic conditions, the development of a software and hardware complex should be carried out under strict weight and size conditions while minimizing production costs. As was shown in works^45–48^, taking into account the limitations^49–53^, the economic assessment of the work consists not only of internal and external factors, but also of the market’s current state. Most parameters of the current state are associated with the demand of the goods and services on the market. Aluminum in this context is one of the main engines in the automobile and aircraft industries. In the context of transportation of oil, gas, biodiesel^54^ and other components that are directly related to the cost of transportation, a number of economic factors influencing metallurgy can be identified:

1. Prices of metal resources: Prices of steel, iron, aluminum and other metals have a direct impact on metallurgical production. Increasing prices for metals can increase the cost of production and reduce the demand for metallurgical products^1–3,10,16,45–49,55,56^.
2. Demand for metallurgical products: economic growth and the construction industry can lead to an increase in demand for metallurgical products, while a recession in the economy can reduce demand^2–5,7,19,22,28–30,45–49,57–62^.
3. Innovation and technological progress: the introduction of new technologies and processes can increase the productivity and efficiency of metallurgical production, which can have a positive impact on its development^2–5,7,19,22,28–30,57,63–65^.
4. Government policy and regulation: Government regulatory measures such as taxes, legislation and customs duties can influence the stimulation or restriction of metallurgical production^2–5,7,11,12,14,23–25,27,38–44^.
5. .International competition: Metallurgical production is a global market, and competition from other countries can put pressure on local producers^1–5,7,54–56,59–70^.

Thus, within the framework of this study, the development of a software and hardware complex has strict restrictions in terms of weight and size. Since the developed product is tasked with confirming the fundamental possibility of such diagnostics, and not with industrial operation, a number of requirements can be neglected.

It should also be noted that within the framework of this study, systems analysis methods will be used aimed at increasing the accuracy of the results. To carry out a system analysis of aluminum electrolysis, the following methods can be most often used:

1. Flow Diagram: This method consists of constructing a diagram that shows the flow of materials, energy and information in an aluminum electrolysis system. A flow diagram helps to identify the main components of the system and determine the flow of resources between them^11,12,14–24,28–42,57,65,71–76^.
2. System model. The system model can be mathematical, physical or computer. It allows you to study the influence of various variables on the operation of the system and optimize the electrolysis process^18–22,28–35,57,77–79^.
3. Function analysis. This method consists of determining the functions performed by the various elements of the aluminum electrolysis system. Function analysis helps to understand how the system works and what problems or improvements may exist^4,5,7,8,79–85^.
4. Analysis of the system structure. This method involves studying the relationships between the various elements of an aluminum electrolysis system. Structure analysis helps determine which elements are most important for the operation of the system and how a change in one element can affect other elements^19,42,82–86^.
5. Stability analysis. This method involves studying the stability of an aluminum electrolysis system. Stability analysis helps to understand how a system reacts to changes in external conditions and what measures can be taken to prevent failures or accidents^34–36,86–91^.

These systems analysis techniques can be applied to study various aspects of an aluminum electrolysis system and optimize the operation of the process. This work involves data collection and physical monitoring. After obtaining a range of initial data, the results obtained can be processed by any of the above proposed methods. This versatility is achieved by using a spatially distributed sensor. It makes it possible to construct a temperature dependence not from one single measurement location, but from all areas of the research object. Similar works were considered in^92–106^ but were applied to other objects. Thus, the logicality of the chosen research path was confirmed.

## Results

### The development of the program apparatus complex for the electric field measuring

**Figure 2:**
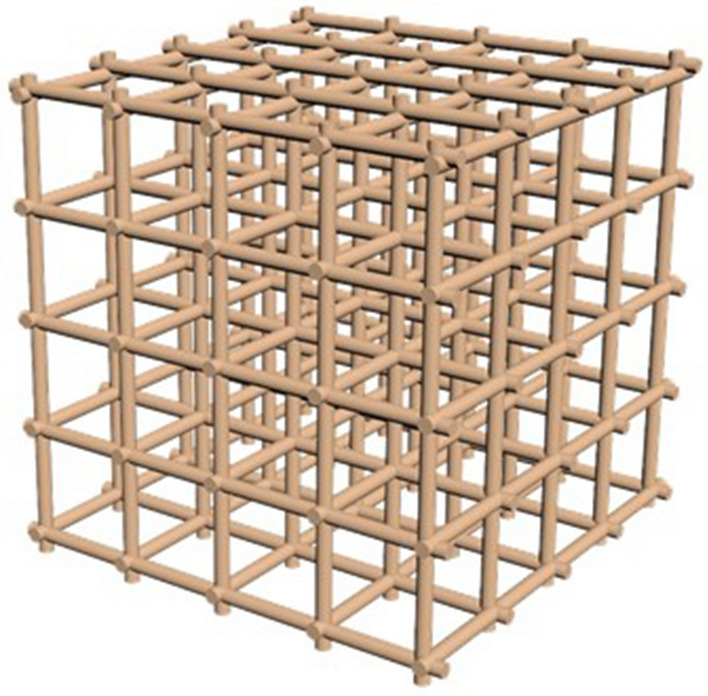
Equilateral grid of the measuring complex.

The main device for measuring the electromagnetic field is the Hall sensor. The principle of operation of the sensor is based on the Hall effect and its initial voltage is directly proportional to the magnetic field strength. Analog sensors make it possible to obtain a differentiated value of the state of the electromagnetic field within the measured range. The A3144 sensor was selected for laboratory testing. To measure the uniformity of the electromagnetic field, the sensors must be located equidistant from each other. For this, a grid is formed, shown in Fig. 2.

**Figure 3:**
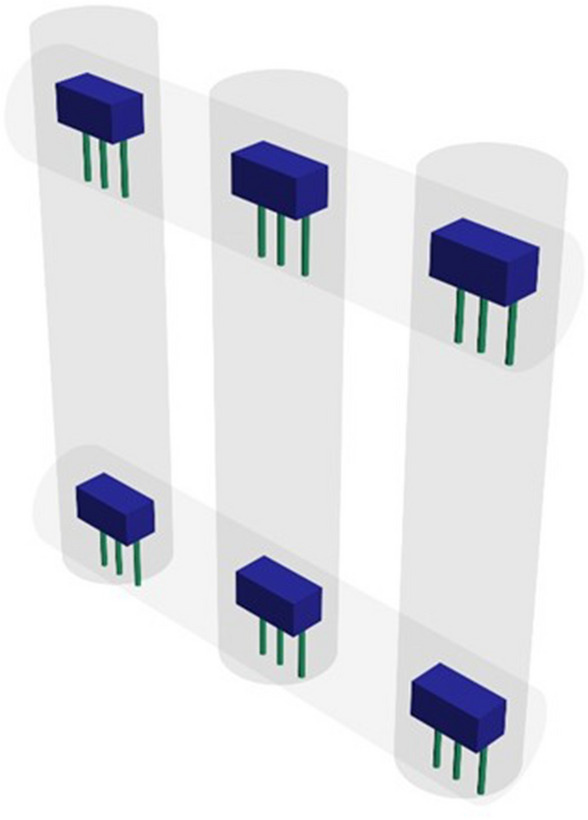
Location of sensors in hollow tubes forming a cube (fragment).

The equilateral grid is a 5 × 5 sensor cube. Thus, there are 25 sensors in the layer, directed perpendicularly to the walls upwards. The sensors are located in hollow tubes that form the walls of the cube (Fig. 3).

**Figure 4:**
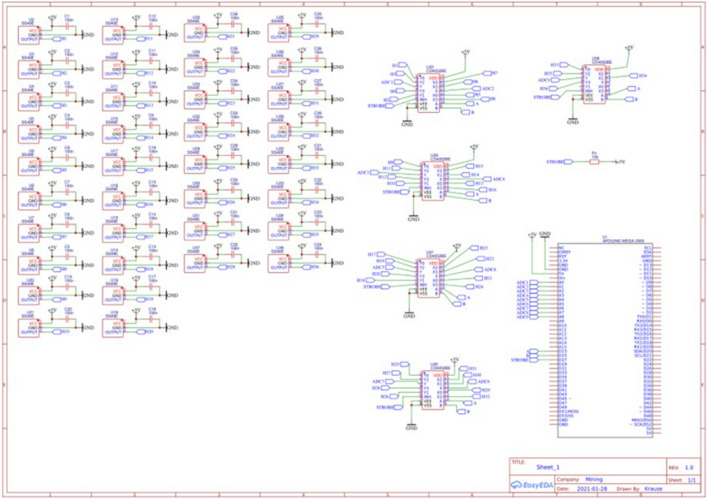
Scheme of connecting Hall sensors to the controller.

Thus, an equilateral grid is formed consisting of four layers of 25 sensors each. Each sensor is connected to an 8-channel multiplexer, and then to the control microcontroller. Then 100 sensors are combined into a single calculation system. For the convenience of building an electronic circuit, all power and ground buses are connected by single conductors. Thus, there is no overload of the current-carrying bars to each sensor. Data buses have an independent supply to the multiplexers. In general, the electronic circuit of each mounting layer looks like shown in Fig. 4.

The software part of the developed program apparatus complex (PAC) consists in the program code executed in C +  + in the Arduini IDE. A feature of the developed program code is the absence of a multi-level connection with the I/O libraries. Since the main problem of sensors is the response time, and for such a large number of them, the delay time will be 6.4 s. The authors made a decision on parallel processing of data from sensors. The microcontroller allows you to process data from 4 channels simultaneously. By setting separators for the data stream received from the controller, we managed to reduce the response time to 1.6 s. A sketch that can be closed into a microcontroller that performs this function is presented below:


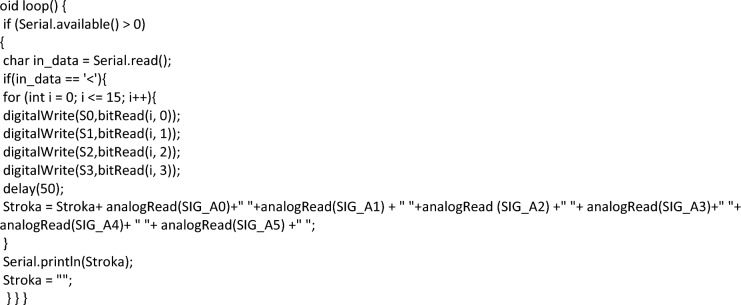


The data rate is set to 9600 kilobits per second. The total amount of input data does not exceed 6000 kilobits per second.

A software module has been developed to process the received data. The module was developed in Borland Developer Studio 2006^107^ in the Delphi programming language. COM + libraries^108^ are used to work with external devices. After connecting the specified libraries, the library model looks like this:


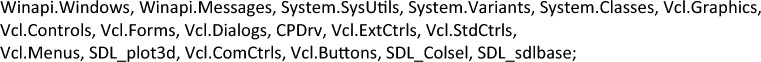


The program code fragment that loads data from COM into the program looks like this:


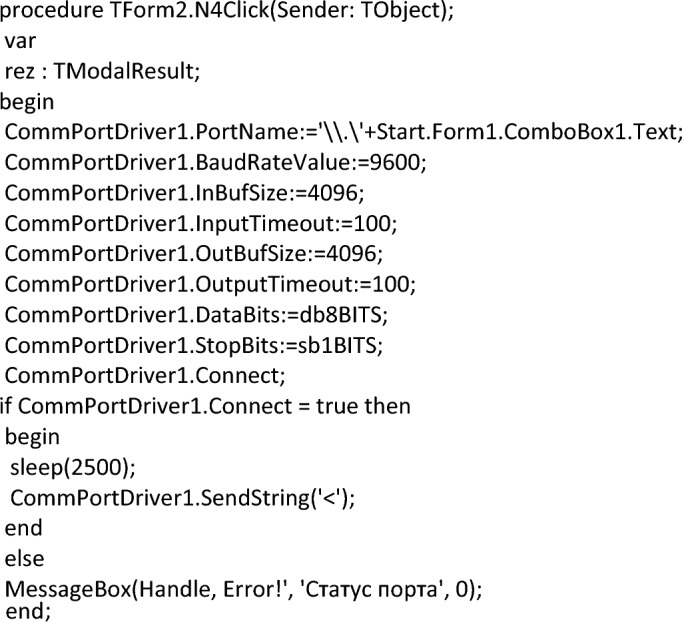


The data is read in a line of 500 characters and with the fixation of the universal separator. Allowing you to identify the boundaries of the data of one sensor from another. In this case, streaming data can easily be represented as a two-column table. In the first column, which indicates the identification number of the sensor (in our case, these are the coordinates of its position), and in the second column, the current readings of this sensor. A fragment of the program code that performs such processing is presented below.


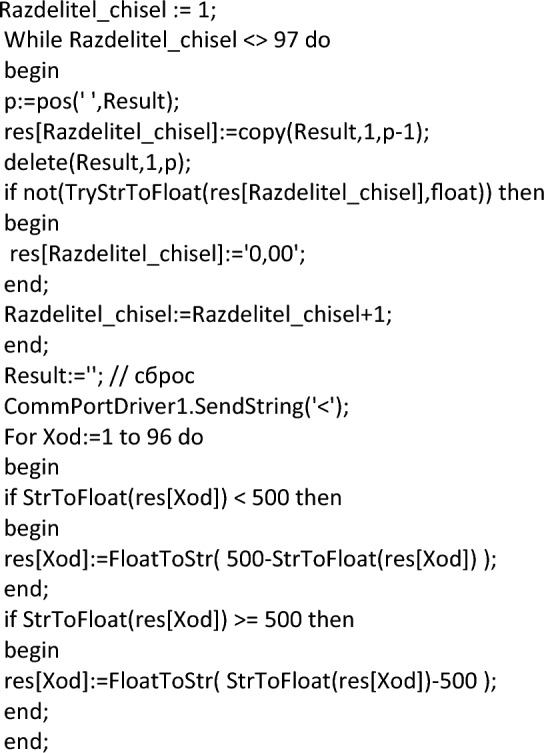


After that, a formula for graphical display the layer values of the sensor is formed. The complete build code is shown below.


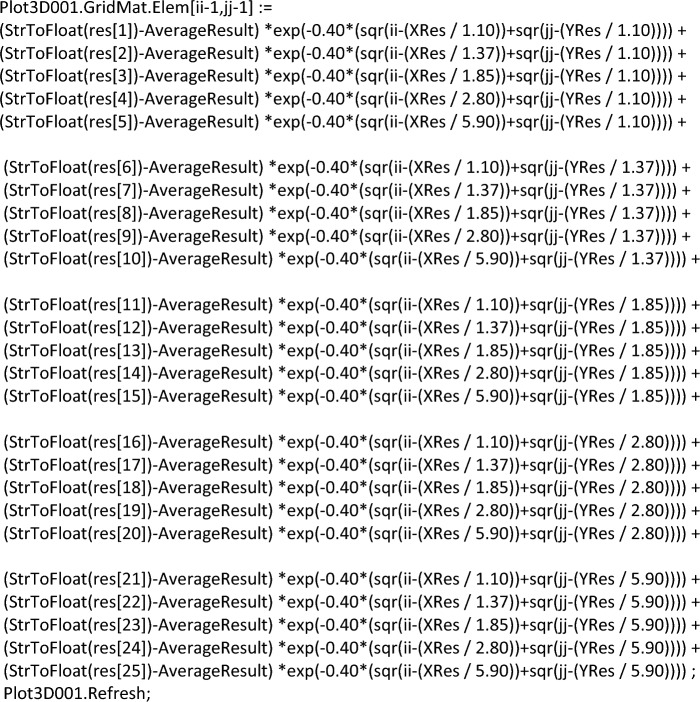


This formula represents five blocks of five lines. This was done for a more informative perception of the grid. If one of the sensors is connected to the wrong port by mistake, this can be easily corrected by correcting the program code. It is also necessary to note the rigid binding of sensors to the coordinate grid. Such a binding will allow us to confirm the correctness of the preparation and collection of data from experiments conducted on the developed device. To display a graphical display, the freely distributed component SDL Component Suite^109^ is used.

**Figure 5:**
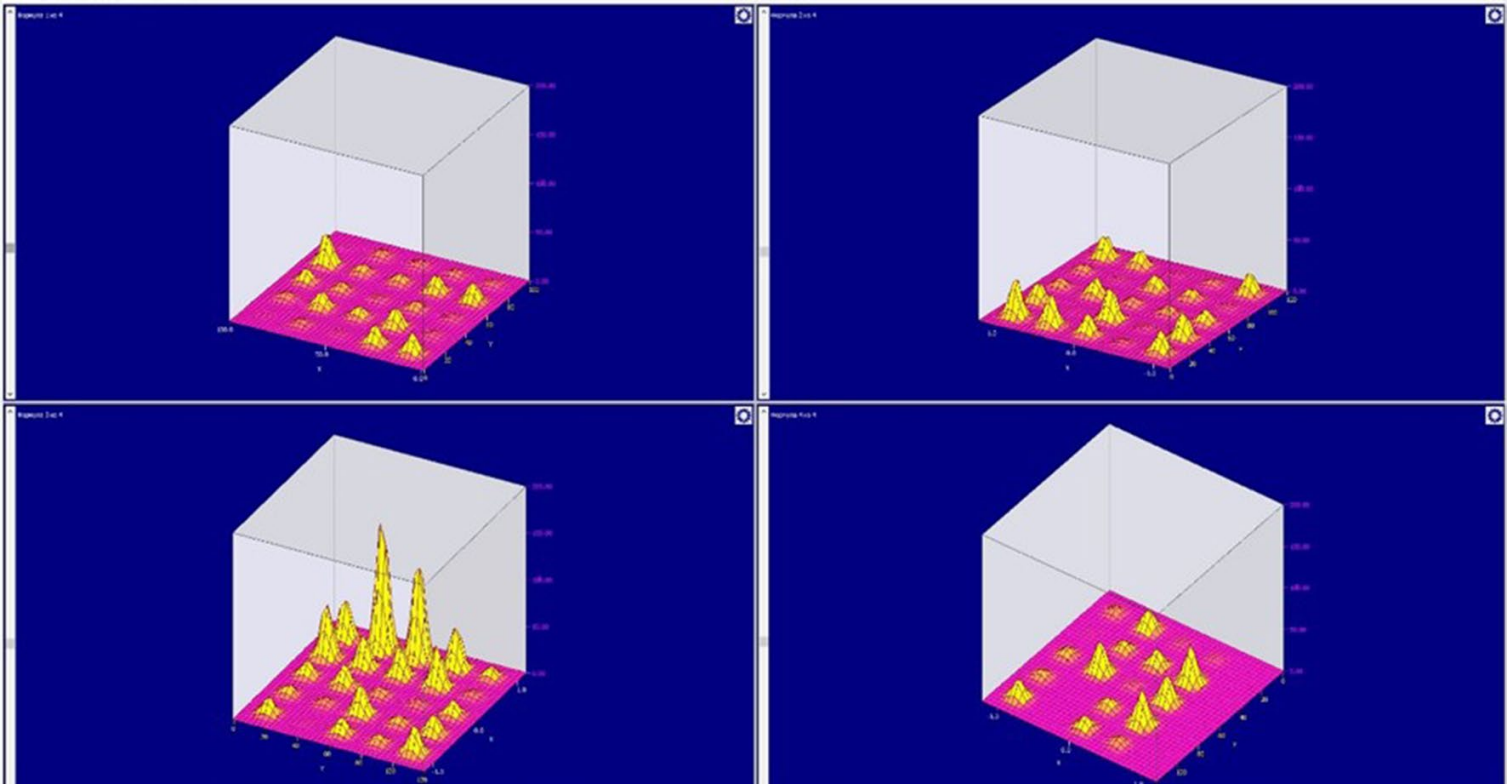
The interface of the developed PAC.

Figure 5 shows the main window of the developed software module. It is divided into four parts, each of which displays the state of the electromagnetic field in the corresponding layer. Accordingly, depending on the location of the object that forms the electromagnetic field, the sensor readings show a larger value (a unit of millivolts).

Thus, within the framework of the first part of the study, a software and hardware complex for measuring the electromagnetic field in laboratory conditions was developed. The task of this complex is to study the generated electromagnetic field and its dynamic characteristics. It is proposed to conduct a number of experimental studies aimed at the analysis of dynamically changing electromagnetic fields.

## Experiments

As an object of study, we consider a working layout of a low-power electrolyzer. Technical characteristics of the electrolyzer are presented in Table 1.

**Table 1:** Technical characteristics of the object of study.

Parameter	Value
Bathtub dry weight	6600 kg
The mass of the filled bath	9300 kg
Bath dimensions (L × W × H)	4378 × 1108 × 1000 mm
Usable bath volume	2, 4 m^3^/h
Pump capacity	2–3, 8 m^3^/h
Electrode power supply	6 V, 2400 A
Control system:	Digital with smooth adjustment of voltage and current on the tires
Closed electrolyte circulation system	Closed electrolyte circulation system with safety overflow, one main circulation pump and one backup circulation pump
Power consumed from the mains 380	1.8 kW
Type of contact bar	Copper

**Figure 6:**
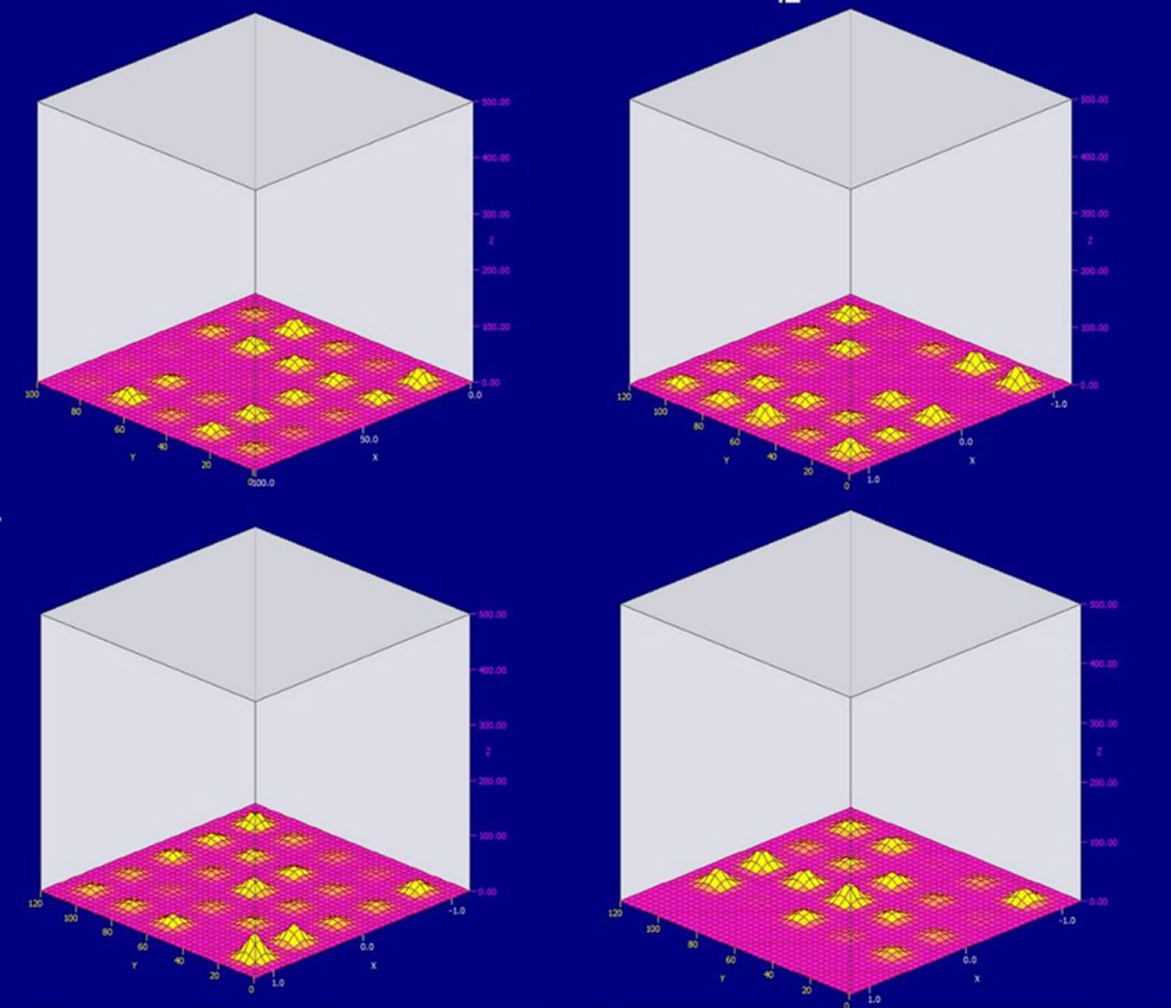
Graphs of the layer-by-layer propagation of the electromagnetic field formed by the first anode (on the graph on the x and z axes, spatial coordinates are indicated, along the y axis the values of the sensor readings are plotted).

Spatially distributed sensors of the electromagnetic field were installed in the area of the anode. The data obtained from the developed device is shown on the Fig. 6.

It is worth paying attention to the fact of the electromagnetic field uneven formation. This can be caused by two factors. The first is the error of each sensor. This shortcoming is eliminated by calibrating the sensors. The second drawback is the poor quality of the welded seams, which do not provide a uniform current flow to the anode. This drawback is eliminated by collecting statistical data and diagnosing current-carrying tires. At the initial stage of the electrolysis process, the anodes consume maximum power. This can be observed in Fig. 6. From the presented figure, one can observe the maximum deviation in the first layer, moderate in the second, and in the third and subsequent minimum values. This is due to the distance of the anode object from the sensors.

**Figure 7:**
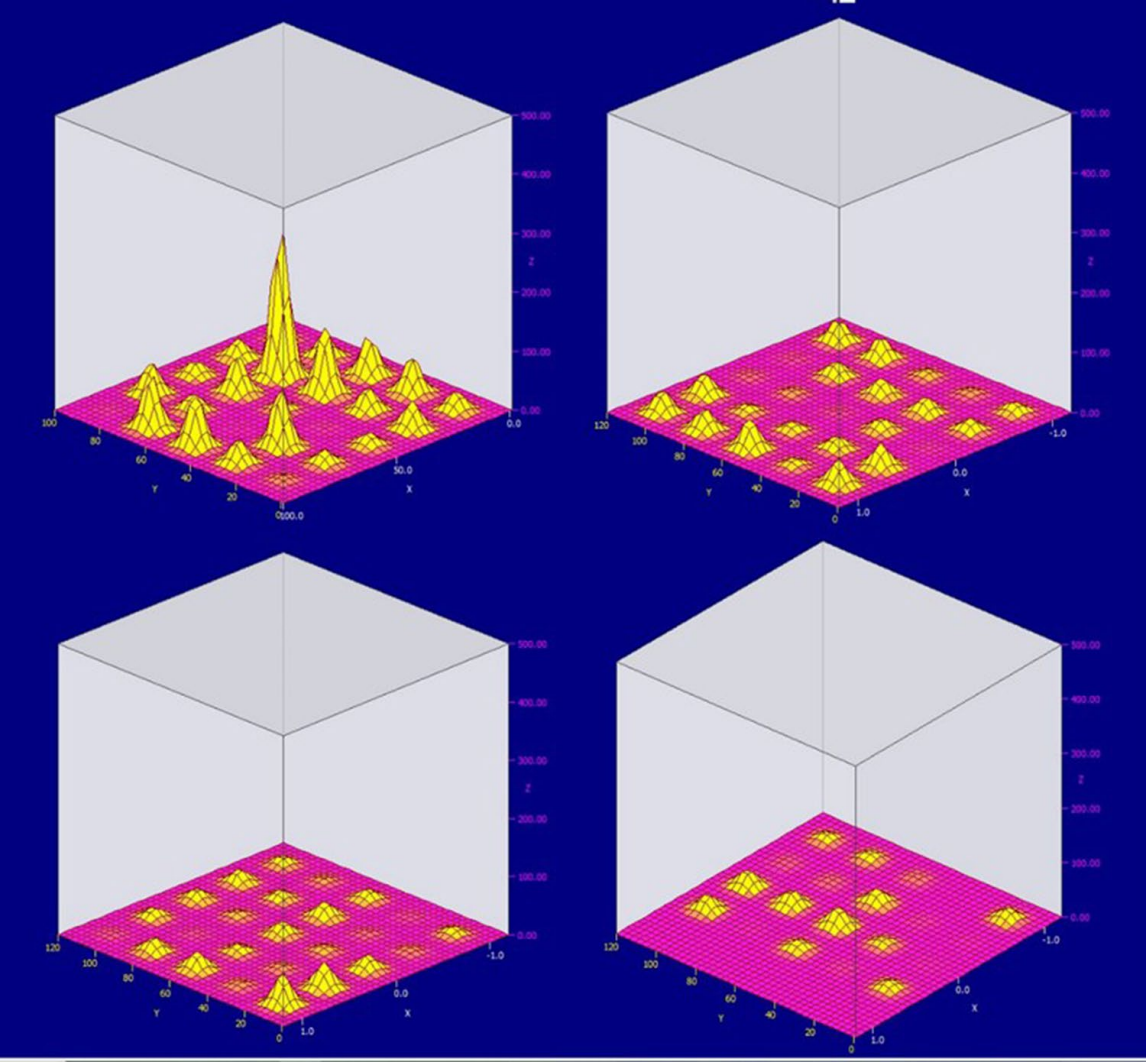
The value of the electromagnetic field between two anodes (1 layer of sensors).

It is also possible to observe a clear sphericity of the formulated electromagnetic field. It is proposed to consider the response of the electromagnetic field during measurements at the junction of two anodes. Figure 7 shows a graphical representation of the electromagnetic field generated by two adjacent anodes (only the first layer of sensors). Let's analyze the readings displayed on the graph.

1. It is necessary to pay attention to the inhomogeneity of the field formed by the anodes. This is due to their uneven burning. Such inhomogeneity causes a difference in the directions of current flow, which leads to the rotation of aluminum in the bath, and as a result, the deformation of the walls of the bath, the destruction of the lining, etc. Also, according to the shape of the electromagnetic field, it can be judged that the anode located on the left burns out in the middle, forming protruding edges on both sides. This will eventually chip off part of the anode and clog the bath.
2. At an equidistant point from two anodes, the value of the electromagnetic field is small. This may be due to insufficient working power of the cell, which can be caused by both uneven burnout of the anodes and initially incorrectly set power of the cell.
3. Extreme points far from the anode have zero readings. This also indicates insufficient power supplied to the anodes and their wear.

Identified defects may also indicate busbar defects and poor or excessive ground contact.

To identify this shortcoming, it is proposed to consider the fourth layer of sensors of the same area.

**Figure 8:**
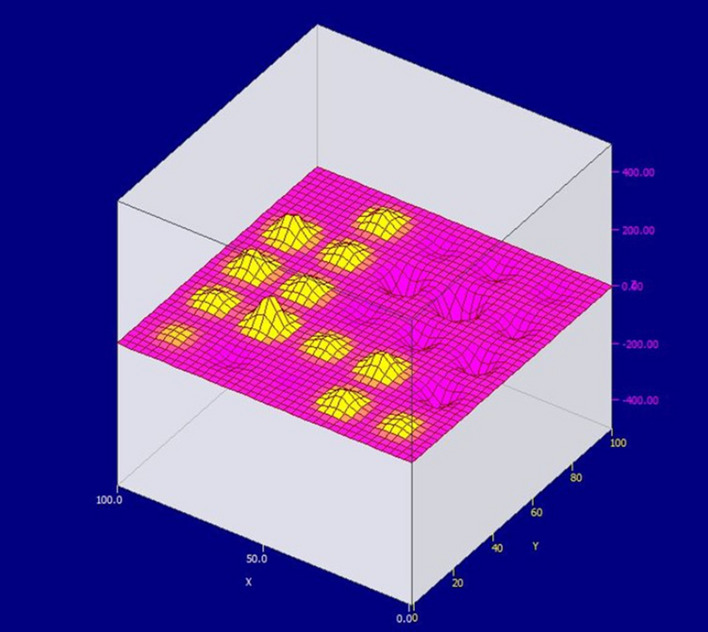
The value of the electromagnetic field between two anodes (4th layer of sensors).

Figure 8 shows that the value of the electromagnetic field changes sign. Which is a sign of a violation of the integrity of the grounding system. It also indicates a low degree of isolation of the circulating currents.

## Discussion

The industrial production of cheap aluminum is important for the industrialized countries economies growth. Within the framework of this study, a number of literary sources of economic, environmental and technical direction were analyzed, which made it possible to evaluate the efficiency of an aluminum production enterprise from an economic point of view. The analysis made it possible to determine the most costly component of the aluminum production process—energy consumption. The electrolyser, as the main consumer of electrical energy, plays the main role in it. The electrolysis process is not an energy efficient process due to the influence of a huge number of factors. As part of this study, the authors developed a device—a spatially distributed sensor of the electromagnetic field. With its help, a number of studies were carried out at a laboratory facility for the production of aluminum. Within the framework of this study, violations of the structural integrity of the electromagnetic field were revealed. These violations could be detected only with the help of the developed device. Since at most enterprises there is a spot measurement of electromagnetic intensity. The developed software and hardware complex allows evaluating the entire structure of the electromagnetic field, pointing out specific shortcomings.

A special place in this study is occupied by the interpretation of the results obtained. It is important to note that the non-stationarity and inhomogeneity of the field makes it possible to point out a specific drawback of the cell element under study or its entirety.

Having obtained these results, shown in Fig. 6, it is possible to replace the burned-out anode at the moment when it burned out, and not when the time for its replacement has come according to the regulatory documentation. It should be noted that, depending on the rate of aluminum production, the anodes burn out unevenly, and in each cell the service life is determined based on either the service life or the difference in the current drop. The proposed hardware-software method will allow you to accurately determine the life of the anode.

The inhomogeneity of the field indicates eddy currents and rotation of the aluminum in the hearth. Using the results of this measurement, one can indirectly judge the direction of rotation. To provide measures the field’s stabilizing and prevent rotation. In industrial conditions, this will significantly increase the operating time of the cell, as it makes it possible to eliminate one of the main factors of its destruction.

Using of the developed software and hardware complex will significantly expand the functionality of diagnostics of industrial electrolyzers. At the same time, a number of shortcomings of this device should be noted. The main one is accuracy. To ensure the given accuracy, it is necessary to use a large number of sensors. If there are not enough of them, the purity of discretization will be too large to determine specific shortcomings. And the use of sector diagnostics will not reflect the dynamics and can only be used for local measurement, which can be eliminated by increasing the number of sensors (measuring points), which will affect the final cost of the product. However, since the device is not disposable and is intended only for diagnostics, and not for continuous monitoring, the significance of this drawback is minimal.

At the same time, it is necessary to note the ways of the developed device further improvement. The works^110–122^ indicate ways to increase the accuracy and industrial adaptation of the developed device. This direction will be the result of authors’ further research.

## Conclusion

In the context of the economic crisis and the deterioration of the environmental situation, production processes that combine the low cost of the product and its high quality are becoming increasingly important. It can be achieved only by creating an environmentally friendly and cost-effective production. The considered economic, environmental, state factors^123^ affecting pricing show the need for a deep analysis of the factors affecting the product as the final product. For the technological process of aluminum production, this is electricity. Also the green energeticimprovement dimension can be considered as a possible implementation area^124^. Within the framework of this study, the authors developed a software and hardware complex for diagnosing the electromagnetic field of the electrolytic cell. A number of studies were carried out on a laboratory unit for the production of aluminum. It should be noted that based on the results of the work, a patent was issued for the invention “Device for diagnosing an electromagnetic field”^58^. Having demonstrated the possibilities of measuring and interpreting the results obtained, a direction was presented to increase the economic efficiency of the electrolytic cell.

## Data Availability

The datasets generated and/or analysed during the current study are available in the «SR Data» repository («Arduino» code for hardware, «Program» calculation code, «Result» additional result’s data), (https://drive.google.com/drive/folders/1_d_m31sKwbeOaSHT-JqZuE8YtNKiWBwd?usp=sharing).
